# Deubiquitinases in cancer

**DOI:** 10.18632/oncotarget.3671

**Published:** 2015-05-07

**Authors:** Rongbin Wei, Xiaodong Liu, Weixin Yu, Tianshu Yang, Wenping Cai, Junjun Liu, Xiao Huang, Guo-tong Xu, Shouliang Zhao, Jianhua Yang, Shangfeng Liu

**Affiliations:** ^1^ Department of Ophthalmology, Shanghai Tenth People's Hospital, Tongji University School of Medicine, Shanghai 200072, P. R. China; ^2^ Department of Neurosurgery, Huashan Hospital, Fudan University, Shanghai 200040, P. R. China; ^3^ Department of Stomatology, Huashan Hospital, Fudan University, Shanghai 200040, P. R. China

**Keywords:** deubiquitinases, cancer, ubiquitylation, inhibitor

## Abstract

Deubiquitinases are deubiquitinating enzymes (DUBs), which remove ubiquitin from proteins, thus regulating their proteasomal degradation, localization and activity. Here, we discuss DUBs as anti-cancer drug targets.

## INTRODUCTION

Posttranslational modification of proteins by ubiquitin is a key regulatory event, and the enzymes catalyzing these modifications have been the focus of many studies. Deubiquitinating enzymes (DUBs), which mediate the removal and processing of ubiquitin, might be functionally important but are less well understood. Approximately 100 human DUBs have been identified, over 90% of which are cysteine-proteases whose catalytic sites contain conserved cysteine (C), histidine (H) and aspartate (D) residues [1]. By regulating the ubiquitin system, DUBs play important roles in multiple biological processes including the cell-cycle, DNA repair, chromatin remodeling, and a wide range of signaling pathways.

Ubiquitylation is reversible process by which ubiquitins are attached to proteins, either singly or in chains. The ubiquitination pathway includes ubiquitin-activating (E1s), ubiquitin-conjugating (E2s) and ubiquitin ligase enzymes (E3s), ultimately responsible for the conjugation of ubiquitin to protein substrates (for reviews see [2, 3]). Ubiquitin can be attached to substrate proteins as a single moiety or in the form of polymeric chains in which successive ubiquitin molecules are connected through specific isopeptide bonds. These bonds can be formed on any of the eight primary amines of the ubiquitin molecule (linear/amino (N) terminus/M1, K6, K11, K27, K29, K33, K48 and K63) and thus can achieve a remarkable complexity, termed the ubiquitin code, in which the different chain topologies have distinct signaling functions [4]. The removal of ubiquitins or polyubiquitin chains from the target protein is catalyzed by deubiquitinating enzymes (DUBs). Therefore, DUBs reverse the function of E3 ubiquitin ligases [5]. The human genome encodes approximately 100 DUBs that are subdivided into 5 families based on sequence and structural similarity: ubiquitin-specific proteases (USPs), ubiquitin carboxy-terminal hydrolases (UCHs), ovarian-tumor proteases (OTUs), JAMM Motif Proteases (JAMMs), Machado-Joseph disease protein proteases (MJD) [5].

Ubiquitin (Ub) is a small, highly conserved protein that is added onto primary amino groups of the acceptor protein through an enzymatic cascade involving sequential actions of E1, E2, and E3 conjugating proteins. It is the E3s that are primarily involved in substrate recognition, and, accordingly, there are more E3s than their E1 and E2 counterparts. The E3s can be subdivided into the really interesting new gene (RING) family (~300 in the human genome) and the homologous E6-associated protein carboxy terminus (HECT) family (28 in the human genome) [6]. Both types of E3 link E2 enzymes with substrates, but they differ in that RINGs do not themselves directly transfer ubiquitin, whereas the HECT family forms an intermediate thioester linkage with the ubiquitin C terminus. Although the RING-E2 interaction occurs at some distance from the E2 catalytic site, it is proposed to facilitate ubiquitin transfer from the E2 by an allosteric mechanism [7, 8].

Deubiquitylation and ubiquitylationare dynamic and reversible post-translational modifications that are involved in the regulation of various cellular pathways. For a long time, functional studies of ubiquitylation have focused on the function of ubiquitylating enzymes, especially the E3 ligases, rather than deubiquitylating enzymes (DUBs) that hydrolyze ubiquitinchains. One reason maybe the smaller number of DUBs in the human genome compared to the larger number of E3 ligases, implying broader substrate specificities of DUBs and difficulties identifying the indirect targets. However, recent studies have revealed that DUBs also actively participate in controlling cellular events in cancer. DUBs are also essential for processing ubiquitin precursors and are important for recycling ubiquitin molecules from target protein prior to their degradation and maintaining the free ubiquitin pool in the cell. Here, we will discuss the five different DUB families (USP, UCH, JAMM, OTU, and MJD) and their known biochemical and physiological roles in cancer. In this review, we examine these recent insights, and attempt to provide a comprehensive overview of what is known about this emerging post-translational modification in cancer. In addition, we will propose some directions for future studies.

## UBIQUITIN PROTEASOME SYSTEM-TARGETED ANTICANCER THERAPEUTICS

Covalent attachment of ubiquitin, a highly conserved protein, to a target protein is a means of regulating protein half-life, localization, and activity. Because protein homeostasis is essential for the survival of all cells, but more essential to cancer cells, modulation of individual ubiquitin-proteasome system (UPS) components might present an opportunity for therapeutic targeting. Consequently, many compounds with proteasome inhibitory activity have been developed, including bortezomib (Velcade; Millenium Pharmaceuticals), which is a synthetic dipeptide boronic acid that reversibly inhibits the chymotrypsin-like activity of the 20S enzymatic core of the proteasome and induces apoptosis in several malignancies. Velcade is approved by the U.S. Food and Drug Administration (FDA) for the treatment of patients with mantle cell lymphoma and multiple myeloma, even those resistant to doxorubicin, mitoxantrone, melphalan, and dexamethasone, and is commonly used in combination with many of these agents [9]. Amplified protein synthesis (immunoglobulin) in many myeloma cells might underlie their clinical sensitivity to bortezomib and other proteasome inhibitors, because solid tumors do not have a similar commitment to elevated protein synthesis and are not clinically responsive to these drugs. This narrow therapeutic application, combined with some toxicity (sensory neuropathy), might be circumvented by novel proteasome inhibitory molecules [9].

## DEUBIQUITINASES AS EMERGING TARGETS FOR ANTICANCER THERAPEUTICS

Targeted the inhibition of ubiquitin-conjugating enzymes and ligases could offer another therapeutic modality. In addition, screening analyse of ubiquitin ligases reveals G2E3 as a potential target for chemosensitizing cancer cells [10]. Inhibition of NEDD8-related E1 enzyme by MLN4924, E2 enzyme hCdc34 by CC0651, and E3 ligase MDM2 by RITA (NSC652287) and MI-219 reflects this ongoing effort [11]. Deubiquitinases (DUBs) are another class of emerging anticancer target that regulate specific substrate proteins by reversing their ubiquitination through the hydrolysis of isopeptide or a-peptide bonds linking ubiquitin to the target protein [12]. If the target protein is an oncogene, the associated DUB might stabilize its cellular expression, which supports the identification of DUB inhibitors that could reestablish normal protein turnover, location, or activity [12]. Auranofin (Aur) inhibits proteasome-associated deubiquitinases (DUBs) UCHL5 and USP14 rather than the 20S proteasome; inhibition of the proteasome-associated DUBs is required for Aur-induced cytotoxicity; and Aur selectively inhibits tumor growth in vivo and induces cytotoxicity in cancer cells from patients with acute myeloid leukemia [13]. This approach might also avoid the deleterious side effects associated with direct targeting of the proteasome. Genetic and/or functional analysis have revealed DUBs in the category of cancer-associated proteases, and their unique biochemical structures have made them desirable targets for anticancer therapies. In recent studies, more than 40 DUBs have been involved in cancer directly or indirectly. These numbers are not surprising, considering the various critical cellular functions regulated by different DUBs and the diversity of substrates used and regulated by them. A comprehensive list of DUBs altered in different cancers is provided in Table 1.

**Table 1:** Mammalian DUBs and Their Reported Functions

Name	Substrate(s)	Process	Tissues	Remarks	Mouse models	References
**UCHs**						
UCH-L1	Unknown	Advanced tumour stage	Breast cancer	Homodimer has E3 activity; mutant mice display ataxia		[118]
BAP1	H2A	ccRCC development	Renal tumorigenesis, various cancers	tumor suppressor	one allele of Bap1, BAP1-deficient cells	[60, 119]
UCH-L5	Unknown	apoptosis/cell death	tumor cells	Binds to proteasome		[120]
**USPs**						
CYLD	TRAF2/6, NEMO TRAF2, TRAF6, NEMO, TRPA1, Tak1, Lck, Bcl3, Dvl	NF-κB and JNK Pathway	Cylindromatosis of the scalp, trichoepithelioma of hair follicles, colitis, hepatocellular carcinoma	Familial tumor suppressor (cylindromatosis)	CYLD-deficient mice exhibit a strong increase in the incidence of tumors in a colitis-associated cancer, mice lacking Cyld are prone to chemically induced skin tumors	[81, 121, 122]
USP1	FANCD2PCNA	DNA repair	Cell lines	Tumor promoter	USP 1-deficient mice with cross-linker hypersensitivity, a Fanconi anemia phenotype and hematopoietic stem cell defects	[44, 123, 124]
USP2a	Fatty acid synthase	Fas/p53, NF-kB, Myc	Prostate cancer, glioma	Circadian-regulated tumor promoter	Transforming gene in NIH3T3 cells injected into athymic mice	[73, 125–127]
USP3	Unknown	DDR	Increased mRNA levels reported in bladder, brain and prostate cancers; reduced levels reported in leukemia and colon cancers	Cancer associated		[128–130]
USP4	Unknown	TGFb, NF-kB, Wnt, p53	Can transform NIH3T3 cells, can induce tumorigenesis in athymic nude mice. Increased expression in human small cell and adenocarcinoma lung tumors and metastatic breast carcinomas	Transforming activity Oncogene	Transforming gene in NIH3T3 cells injected into athymic mice	[89, 105, 131, 132]
USP5	Unknown	p53, DDR	Melanoma, glioblastoma	Cancer associated		[24, 76, 133, 134]
USP6	Unknown	Remodeling NF-kB	Aneurymal bone cysts	Transforming activity; rearrangements and fusions found in cancer Oncogene	USP6 induces formation of tumors that recapitulate multiple features of ABC (xenografts)	[135–137]
USP7	HDM2, p53, H2B	p53, PI3-K, PTEN, FOXO4	Myeloma, prostate cancer, neuroblastoma, gliomas	Binds herpes virus proteinVmw110 Tumor promoter	Decrease in colon tumor size following USP7 silencing (xenografts)	[22, 23, 138, 139]
USP8	NRDP1	Endocytosis, Wnt, hedgehog cytokine receptor signaling	Non–small cell lung cancer	Oncogenic fusion withp85-PI3K tumor promoter		[140–143]
USP9X	β-catenin, epsins, AF-6	TGFb, Mcl-1, ERG, AGS-3ITCH, Wnt, Notch signaling, endocytosis	Human lymphoma, myeloma, ductal, colon, prostate and small-cell lung adenocarcinomas glioblastoma, medulloblastoma, Mouse pancreas ticadenocarcinoma model	Tumor promoterTumor suppressor	Tumor growth delay following USP9x silencing in combination with ABT-737 treatment (xenografts)	[112, 144–148]
USP9Y	Unknown	Spermatogenesis		Mutants associated with azoospermia		[149]
USP10	Unknown	cMyc, p53	Renal cell carcinoma	Tumor suppressor; Tumor promoter		[74, 150–152]
USP11	BRCA2	DDR, NF-kB	Higher recurrence rates and poor survival outcome in breast cancer	Interacts with Ran BPM Tumor promoter		[153–156]
USP14	Unknown	Synapse functionWnt	Colorectal cancer, non–small cell lung cancer	Mutant mice develop ataxia; binds to proteasome Tumor promoter		[157–159]
USP15	RBX1	NF-kB Wnt	USP15 gene is found amplified in human breast and ovarian tumors, and in glioblastoma	Tumor promoter		[160, 161]
USP16	H2A?	Chromosome condensation?RUNX fusion		Oncogene		[162–164]
USP17	Unknown	GTPase subcellular localization and cell motility, G1/S cell-cycle checkpoint	Breast cancer NSCLC distal metastases; metastatic lung cancer	Tumor promoter	Decrease of breast tumor size following DUB3 silencing (xenografts)	[31, 156, 165]
USP18	Unknown	JAK-STAT signaling, immunity, brain functionNF-kB	AML	ISG15-specific; mRNA is induced by IFN-α and IFN-βCancer associated		[166, 167]
USP19	Unknown	ERAD pathway	Breast and prostatecancer	Cancer associated		[168, 169]
USP20	DIO2?	Thyroid hormone metabolism, hypoxia signaling NF-kB	Von Hippel-Lindausyndrome	Interacts with PvhlOncogene		[170, 171]
USP21	Unknown	NF-kB	Metastatic urothelialcarcinoma	Cleaves Ub and NEDD8but not SUMOCancer associated		[172]
USP22	Unknown	C-Myc	Increased expression in salivary ductcarcinoma, papillary thyroid carcinoma, non–small cell lung carcinoma, oral squamous cell carcinoma and colorectal cancer and poor prognosis inglioblastoma. Novel prognostic marker for improving treatment efficiency for patients with glioblastoma.	Tumor promoter		[173–178]
USP25	Unknown	ERAD pathway	Overexpressed inhuman breast cancer	Cancer associated		[150, 179, 180]
USP26	Unknown	Spermatogenesis		testis specific		[181–183]
USP29	Unknown	p53		Cancer associated		[77, 184]
USP32	Unknown		Overexpressed in breast cancer	Tumor promoting		[185, 186]
USP33	HIF1-α DIO2?	Hypoxia signaling		Interacts with PvhlOncogene		[187, 188]
USP42	Unknown	p53, RUNX fusion gene	AML	Oncogene		[131, 189]
USP50	Unknown	G2/M Checkpoint	AML	Cancer associated		[35]
DUB3	Unknown	G2/M Checkpoint	Breast cancer	Cancer associated		[36, 190]
**MJDs**						
Ataxin-3	Unknown	Gastric carcinogenesis and development	Gastric cancer, lung cancer cells	Cancer associated	Mouse ataxin-3 functional knock-out model.	[191–193];
**OTUs**						
A20	RIP	NF-κB signaling	Tumor cells	Tumor suppressor		[85, 194]
JAMMs	Unknown					
POH1	19S proteasome	Proteasome	Tumor cells	Cancer associated		[195].
CSN5	Cullins	Wnt/β-catenin activation	Tumor cells colorectal cancer cells	Cancer associated		[196, 197]
BRCC36	Unknown	G2/M checkpoint signaling	Breast cancer	Enhances BRCA1/BARD1E3 ligase activity		[198]

## DEUBIQUITINASES IN CANCER

It is now well known that DUBs have significant impacts on the regulation of multiple biological processes such as cell-cycle control, DNA repair, chromatin remodeling and several signaling pathways that are frequently altered in cancer [5, 14] (Table 1). As a result, different DUB functions are directly and indirectly involved in tumorigenesis.

### DUB-associated mutations in cancer

Recurrent mutations of DUBs are rare in cancer with the exception of *CYLD*. Germline mutations of the tumor-suppressor gene CYLD are prevalent in familial cylindromatosis, a genetic condition that leads to a predisposition for developing multiple skin tumors [15]. A well-known chromosomal translocation involving a USP is the fusion of the promoter of CDH11 to the full-length USP6 gene leading to upregulated transcript levels of USP6 [16]. The USP1 amino acid motif 420–520 is necessary and sufficient for UAF1 binding. USP1 autocleavage can occur in cis, and can be altered by a cancer-associated mutation [17].

### Development of small molecule inhibitors against DUBs

DUBs, as therapeutic targets in cancer and other diseases [14, 18, 19], and DUBs play important roles in recycling ubiquitin monomers to prevent proteasomal degradation of proteins tagged with ubiquitin, and in trimming ubiquitin from tagged proteins [20, 21]. DUBs have been described as a class of anticancer targets; inhibitors of these enzymes were discussed above in the Proteasome section. It is the second function, sparing of target proteins by the removal of conjugated ubiquitin, that has made DUBs attractive targets for cancer and other diseases [20]. In the past decade, tool compounds and/or preclinical development candidate small molecule DUB inhibitors have been reported; these compounds were identified as inhibitors of several DUBs and have a range of reported selectivities with respect to other DUBs and other cysteine proteases [22–24]. Some examples of these inhibitors are given below; their story is a chronicle of the state of DUB-based anticancer drug development up to the present time (Table 2).

**Table 2:** Experimental DUB inhibitors

DUBs	Compound	References
**USP1**	Pimozide; GW7647; ML323 and ML323 (70)	[11, 199]
**USP5**	WP1130, EOAI3402143 (G9); Vialinin A	[24, 28, 200]
**USP7**	HBX41, 108, HBX19,818 ; P0050429 and WO2013030218	[22, 23, 201]
**USP8**	HBX 41,108	[202]
**USP9X**	WP1130;EOAI3402143 (G9)	[24]
**USP11**	Mitoxantrone	[24]
**USP14**	WP1130, b-AP15, Auranofin	[24, 120, 203, 204]
**UCH37**	WP1130	[24]
**UCH-L1**	UCH-L1inhibitor1	[205]
**UCHL5**	b-AP15, Auranofin	[203, 204]
**DUB (pan)**	12PGJ2; PR-619	[206, 207]

### Cell-cycle regulation

Many facts have revealed that DUBs play a critical role in cell-cycle progression since several DUBs are integral components of the core cell-cycle machinery and cell-cycle checkpoints. Functional studies have demonstrated that USP28 play an important part in regulating the stability of c-Myc, a central modulator of cell growth, proliferation and apoptosis [25]. Some DUBs such as CYLD, USP5, USP13, USP15, USP17, USP37, USP39 and USP44 are essential regulators of events occurring in mitosis. As a consequence, CYLD is crucial for timely entry into mitosis through the regulation of polo-like kinase 1 [26, 27]. USP5 in melanoma suppressed cell growth by reinforcing the S/G2-M checkpoint, enhancing extrinsic caspase activation through modulation of p53 and FAS levels and amplifying the apoptotic activity of kinase inhibitors [28]. Moreover, USP13 is recruited by the ubiquitin-recognition protein Ufd1 to antagonize anaphase-promoting complex (APC/C)-Cdh1-mediated ubiquitylation of Skp2, thereby accumulating the cyclin-dependent kinase inhibitor p27 and causing a concomitant cell-cycle delay [29]. USP15 stabilizes newly synthesized RE1-silencing transcription factor (REST) and rescues its expression at the mitotic exit [30]. Moreover, USP17 was differentially expressed during the cell cycle, and it was discovered that USP17-knockdown caused a G1 cell cycle block and inhibited the proliferation of tumor-derived cell lines by attenuating GTPase signaling, and that USP17 is tightly regulated during cell division and its expression is necessary to coordinate cell cycle progression [30, 31]. Deubiquitinase USP37 is activated by CDK2 to antagonize APC(CDH1) and promote S phase entry [32]. USP39 is essential for mitotic spindle checkpoint integrity and depletion of USP39 in mRNA processing contributes to a specific reduction in Aurora B-mRNA levels [33]. USP44 prevents the premature activation of APC/C by stabilizing the APC/C-inhibitory Mad2–Cdc20 complex through deubiquitylation which contributes to the generation of the switch-like transition controlling anaphase entry [34]. In contrast, USP50 is involved in the G2/M checkpoint and serves as a regulator of HSP90-dependent Wee1 stability to suppress entry into mitosis [35]. Furthermore, USP17L2 (known as DUB3) deubiquitylates and is responsible for the stabilization of Cdc25A to promote oncogenic transformation in human breast cancers. In addition, USP17L2 is an example of a transforming ubiquitin hydrolase that subverts a key component of the cell cycle machinery [36]. Moreover, USP2 directly interacts with cyclin D1 and promotes its stabilization by antagonizing ubiquitin-dependent degradation. In addition, targeting USP2 is an effective approach to induce growth suppression in cancer cells with aberrant overexpression of cyclin D1 [37]. USP19 regulates cell proliferation and p27(Kip1) levels in a cell context-dependent manner through both E3 ligase KPC1-dependent and KPC1-independent mechanisms [38]. Likewise, USP7 (a mdm2 regulator of *p53* function) is essential in cell proliferation and differentiation, through its regulatory activity on phosphatase and tensin homolog and FOXO localization [39, 40]. Moreover, CSN5/Jab1 acts as a modulator of the mammalian cell cycle, preventing senescence and endocycle as well as the proper progression of the somatic cell cycle [41]. Finally, BAP1 forms complexes with the transcription factors Yin Yang 1 (YY1) and host cell factor 1 (HCF-1), and controls cell-cycle progression at G1/S by co-regulating transcription from HCF-1/E2F-governed promoters [42, 43].

### DNA damage repair

The link between DNA damage repair and tumor development is demonstrated by the increased cancer rates reported for those disorders involving deficient DNA repair mechanisms, such as Fanconi anemia. USP1 is responsible for DNA damage repair by regulating Fanconi anemia protein (FANCD2) through deubiquitylation and the subsequent stabilization of checkpoint kinase 1 (CHK1) [44, 45]. Furthermore, USP1 controls proliferating cell nuclear antigen ubiquitylation, a safeguard factor against error-prone DNA translesion synthesis that is ubiquitylated and responsible for genotoxic stress [46]. As a result, USP1 forms a complex with U2 small nuclear ribonucleoparticle auxilliary factor 1 and promotes double-strand break repair through homologous recombination [47]. Moreover, DUB UCHL5 regulates DNA double-strand breaks (DSBs) resection and repair by homologous recombination through protecting its interactor, NFRKB, from degradation [48]. Dub3 controls DNA damage signaling by directly deubiquitinating H2AX [30]. Other DUBs are implicated in the regulation of DNA repair. Thus, BRCC36, Dub3, USP3, USP16, USP44 and OTUB1 participate in regulating the RNF8/RNF168 pathway of double strand breaks repair [49], and USP5 links the suppression of p53 and FAS levels in melanoma to the BRAF pathway [50]. USP11 is involved in the cellular response to mitomycin C-induced DNA damage within the BRCA2 pathway signaling [51]. USP20 is a novel regulator of ATR-dependent DNA damage [52]. USP20 serves as a novel regulator of ATR-dependent DNA damage checkpoint signaling through the deubiquitination and stabilization of claspin and enhances the activation of ATR-Chk1 [53]. Moreover, USP28 is required to stabilize Chk2 and 53BP1 in response to DNA damage; both USP28 and Chk2 are required for DNA-damage-induced apoptosis, and they accomplish this in part through the regulation of the p53 induction of proapoptotic genes such as *PUMA* [54]. USP34 promotes a feed-forward loop to enforce ubiquitin signaling at DNA double-strand breaks (DSBs), and highlights the critical roles of ubiquitin dynamics in genome stability maintenance [55]. Finally, USP47 has been identified as the enzyme responsible for the deubiquitylation of the base excision repair DNA polymerase (Polβ), thus playing an important role in regulating DNA repair and maintaining genome integrity [56]. In the last decade, protein alterations, such as deubiquitination, have emerged as key modifications in the control of DNA damage response (DDR) signaling.

### Chromatin remodeling

Some DUBs interact with histones, predominately H2A and H2B, the post-translational modifications of which control chromatin structure dynamics and gene expression, which are processes that are frequently altered in cancer. To the best of our knowledge, there are at least eight DUBs that can deubiquitylate histones including BAP1, USP3, USP7, USP16, USP21, USP22, MYSM1 and BRCC36 [57–60]. Both H2A and H2B are deubiquitylated by these DUBs, although H2A is preferentially targeted [61]. MYSM1, USP7, USP22 and BRCC36 are part of the 2A-DUB, polycomb-repressive complex 1, SAGA and BRCA1-A multisubunit complexes, respectively [62–65]. However, studies have not identified USP3 and USP16 as being involved in any of these complexes, suggesting that their chromatin-regulatory mechanisms might be different. Apart from histones, gene transcription can be regulated by the deubiquitylation of other chromatin-associated substrates. Therefore, USP22 regulates the protein stability of telomeric-repeat binding factor 1 [66]. Nevertheless, MEL18 and BMI were deubiquitylated by USP7 and USP11, two chromatin-bound components of polycomb-repressive complex 1 complex components that influence the transcriptional regulation of p16INK4a [65]. Furthermore, UCHL5, the activation of which is crucial for the proteasome, also interacts with the human lno80 chromatin-remodeling complex [67]. Finally, as a result, BAP1 deubiquitylates the chromatin-related protein host cell factor 1, which regulates transcription by linking histone-modifying enzymes to a subset of transcription factors [43].

### Signaling pathways

Recent studies have recognized the fact that it is more important to focus on signaling pathway rather than on individual genes altered in cancer [68]. Therefore, mutations in individual genes associated with the same cancer-relevant signaling pathway have been demonstrated in many tumors and are known to have similar functional effects, providing a wide range of drug targets [69]. Some signaling pathways are recurrently altered in many cancers, as those involving p53, NF-kB, receptor tyrosine kinases (RTKs), Wnt, transforming growth factor-b (TGF-β) IFN and Akt, which are significantly influenced by the activity of DUBs.

### The p53 signaling pathway

p53 is, a tumor suppressor that is well studied because it has critical functions in maintaining cellular homeostasis and is frequently mutated in most tumors [70]. To date, research studies have reported many DUBs that are associated with p53 regulation: USP2, USP4, USP5, USP7, USP10 and USP29. USP7 is involved in the dynamic regulation of the p53-MDM2 pathway by controlling the stability of both p53 and MDM2, a ubiquitin ligase that also contributes to the maintenance of p53 ubiquitylation levels [71, 72]. Therefore, USP7 can be regarded as an oncogene or a tumor suppressor depending on whether it mainly deubiquitylates MDM2 or p53, respectively. Similarly, USP2 influences the stabilization of MDM2, but in contrast to USP7, does not deubiquitylate p53 [73]. USP10 is also associated with regulation of p53 localization and stability, however, unlike USP2 and USP7, it does not interact with MDM2 [74]. Otub1, a DUB from the OTU-domain containing protease family abrogates p53 ubiquitination and stabilizes and activates *p53* in cells independent of its deubiquitinating enzyme activity [75]. Interestingly, USP10 can stabilize both mutated and wild-type *p53*, thus having a dual role in tumorigenesis depending on p53 status. Likewise, USP5 has been identified as a potential target for p53 activating therapeutic agents for the treatment of cancer [76]. Moreover, USP29 deubiquitylates and stabilizes p53 in response to oxidative stress [77]. Furthermore, USP4 interacts directly with and deubiquitinates ARF-BP1, leading to the stabilization of ARF-BP1 and subsequent reduction of p53 levels [78]. Taken together with the finding that USP4-deficient murine embryonic fibroblasts (MEFs) exhibit retarded growth, premature senescence, hyperactive DNA damage checkpoints and resistance to oncogenic transformation, thus suggesting that USP4 is a potential oncogene [78].

### The nuclear factor-kappa B (NF-kB) signaling pathway

It is well-known that the nuclear factor-kappa B (NF-kB) signaling pathway has multiple roles in cancer progression including anti-apoptosis, cell cycle, angiogenesis and metastasis [79]. Several DUBs such as A20, CYLD, MCPIP1, USP2, USP4, USP11, USP15, USP21 and USP34 are involved in NF-kB signaling by acting on several components of the pathway [80]. A20 and CYLD regulate levels of ubiquitin related to TRAF6. Furthermore, CYLD is also associated with the deubiquitylation of TGF-B-activated kinase 1 [81], B-cell CLL/lymphoma 3 (Bcl-3) [82] and mitogen-activated protein kinases [83], whereas A20 promotes the degradation of TRAF2 in lysosomes by means of its own E3 ligase activity [84]. A20 also potentiates the proteasomal degradation of RIPK1 through Lys48 polyubiquitylation, whereas its OUT domain removes Lys63-linked ubiquitin chains of RIPK1, leading to the downregulation of NF-kB signaling [85]. The interactions between CYLD and OTU deubiquitinases with linear linkage specificity (OTULIN) and the linear ubiquitin chain assembly complex (LUBAC) ligase are involved in controlling the extent of TNF-a-induced NF-kB activation in cells by fine-tuning the generation of linear ubiquitin chains by LUBAC [86]. MCPIP1 negatively controls c-Jun N-terminal kinase and NF-kB activity through deubiquitylation of TRAF2, TRAF3 and TRAF6, thus playing an essential role in the regulation of inflammatory signaling [87]. USP2 has been reported as a regulator of TNFα-induced NF-kB signaling, and is required for Ikb phosphorylation, NF-kB nuclear translocation and NF-kB-dependent target genes expression [88]. USP4 also plays an essential role in the downregulation of TNFa-induced NF-kB activation through deubiquitylation of TGF-b-activated kinase 1 [89]. Furthermore, OTUD5 deubiquitylates TRAF3 resulting in diminished type I interferon and interleukin-10 responses [90]. Moreover, USP15 stabilizes IkBa by inhibiting its degradation by the proteasome [1] and USP11 interacts with IkB kinase a, an inhibitor of NF-kB, upon induction by TNFα [1]. USP21 inhibits NF-kB activation through the deubiquitylation of RIPK1 [91]. Likewise, Cezanne suppresses NF-kB nuclear translocation and transcriptional activity by deubiquitylating RIPK1 signaling intermediaries and interacting with DJ-1 [92, 93]. USP34 silencing spared upstream signaling but led to a more pronounced degradation of the NF-κB inhibitor IκBα, and culminated in an increased DNA binding activity of the transcription factor [94].

### RTK signaling pathways

Recent studies have found that the relevance of receptor tyrosine kinase (RTK)-mediated signal transduction pathways (RTKs) in several human tumor types is reflected by multiple abnormalities in RTK-dependent pathways [95]. To date, numerous oncogenic mechanisms are known to interfere with RTK internalization. There are at least five DUBs: USP8, USP17, USP18, AMSH and POH1-that affect the trafficking of RTKs including epidermal growth factor receptor (EGFR), Met and ErbB2. USP8 has an important role in the stabilization of RTKs through deubiquitylation, allowing their recycling to the plasma membrane [96]. USP8 depletion might reflect the loss of ESCRT-0 components which associate with the retromer components Vps35 and SNX1, whereas failure to deliver lysosomal enzymes efficiently might also contribute to the observed block in receptor tyrosine kinase degradation [97]. Furthermore, the endosome-associated AMSH (also known as STAMBP) promotes EGFR recycling at the expense of lysosomal sorting [98, 99]. Moreover, USP18 has been identified as a new regulator of EGFR synthesis by controlling its translation [100]. Recent research has shown that USP18 controls EGFR expression and cancer cell survival depending on the transcriptional activation and mRNA stabilization of miR-7 host genes [101]. Finally, another RNA interference screen has identified POH1 as a regulator of ubiquitylated ErbB2 levels, although it is not associated with its turnover [102].

### The Wnt signaling pathway

The Wnt signaling pathway is required for regulation of embryonic development and is frequently activated in cancer [103]. To date, there are at least eight DUBs involved in this pathway, including CYLD, USP4, USP7, USP14, USP15, USP34, TRABID and OTULIN. In fact, CYLD serves as a negative regulator of Wnt signaling and β-catenin activation by deubiquitylating the cytoplasmic effector Dishevelled (Dvl) [104]. USP4 negatively regulates Wnt signaling by interacting with Nemolike kinase [105] and USP15 promotes β-catenin degradation through the stabilization of adenomatous polyposis coli (APC), a negative regulator of Wnt-mediated transcription [106]. USP7 interacts with RNF220 by stabilizing β-catenin, leading to the RNF220/USP7 complex deubiquitinating β-catenin and enhancing canonical Wnt signaling [107]. USP14 functions as a positive regulator of the Wnt signaling pathway. Tissue microarray analysis of colon cancer has consistently revealed a strong correlation between the levels of USP14 and β-catenin, which suggests an oncogenic role for USP14 via the enhancement of Wnt/β-catenin signaling [108]. In contrast, USP34 serves as a positive modulator of Wnt signaling by inhibiting β-catenin-dependent transcription [109]. Furthermore, TRABID is a DUB that is critically involved in T-cell factor (TCF)-mediated transcription of Wnt genes by deubiquitylating APC [110]. The interaction of HOIP with OTULIN is also involved in OTULIN suppressing the canonical Wnt signaling pathway activation by LUBAC [86].

### The transforming growth factor-β signaling pathway

Transforming growth factor-β (TGF-β) is a multifunctional protein that plays a dual role in oncogenesis by serving as an antiproliferative factor at early stages and promoting epithelial-to-mesenchymal transition at later stages [111]. To date, many DUBs have been reported to be involved in the regulation of the TGFβ pathway, including USP4, USP11, USP15, USP18, USP9X and CYLD. The UCH member UCH37, OTU members A20 and OUTB1 and JAMM/JPN+ member AMSH, are also reported to regulate the TGFβ pathway. USP9X positively regulates TGF-β signaling by deubiquitylating SMAD4 and promoting its association with SMAD2 [112]. USP9X regulates cell polarity and proliferation and modulates their phosphorylation and activation by LKB1 through deubiquitylation of the AMPK-related kinases NUAK1 and MARK4 [113]. Finally, AMSH-LP and UCHL5 promote TGF-β responses through their interaction with inhibitory I-SMADs [113, 114].

### The IFN pathway

The IFN immune system consists of type I, II, and III IFNs, signals through the JAK-STAT pathway, and plays a central role in host defense against viral infection. Posttranslational modifications like ubiquitination regulate diverse molecules in the IFN pathway. USP13 was the first DUB identified to modulate STAT1 and play a role in the antiviral activity of IFN against DEN-2 replication [115]. USP2b negatively regulates IFN-β signaling and the antiviral immune response by deubiquitinating K63-linked polyubiquitin chains from TBK1 to terminate TBK1 activation [116].

### The Akt pathway

USP12, in complex with Uaf-1 and WDR20, directly deubiquitinates and stabilizes the Akt phosphatases PHLPP and PHLPPL resulting in decreased levels of active pAkt. Depleting USP12 sensitizes prostate cancer cells to therapies aimed at Akt inhibition irrespective of their sensitivity to androgen ablation therapy [117].

## DISCUSSION AND FUTURE DIRECTION

Conclusively, there is accumulating experimental evidence that a large number of DUBs play important role in several stages of cancer development and progression. The potential to influence processes including signal transduction, proliferation and apoptosis by affecting ubiquitination and proteasomal degradation of key regulators is both promising and exciting. DUBs are more likely drug candidates than E3 ligases due to the lack of defined catalytic residues in the latter. Most DUBs are cysteine enzymes, which should be easy to use as target drugs, particularly if using compounds containing Michael acceptors. Furthermore, many DUBs show profound changes in their expression levels in different malignant tumors. As a result, together with the recent success of clinically targeting the ubiquitin proteasome system in cancer, DUBs have emerged as appealing targets in the development of novel specific therapies against human malignancies. To date, no DUB-targeted strategies have reached clinical trials and many challenges remain before translating this information into clinical benefits for cancer patients. In addition, DUBs could be targeted for anticancer therapeutics. Early evidence for antitumor efficacy with the currently available DUB inhibitors is more than encouraging and sets the stage for the development of selective, as well as partially selective, small-molecule DUB inhibitors. Moreover, the assignment of oncogenic or tumor-suppressive roles to certain DUBs is still due to the cellular context and further analysis will be required for functional and clinical validation of DUBs as drug targets. Bioavailability is a hurdle that needs to be overcome, but if progress can be made in this area molecules containing functional dienones as well as other types of compounds might be developed into useful cancer therapeutics. Finally, the generation of gain- or loss of- function animal models for selected DUB family members will likely contribute to clarify the relative relevance of individual DUBs and their alterations in tumorigenic progression. Hopefully, our review derived from many studies will provide novel insights into the multiple questions associated with DUBs and lead to the introduction of DUB-targeting therapy in cancer treatments and crucial components of molecular therapies against cancer.
